# Intestinal Syphilis

**Published:** 1916-03

**Authors:** 


**Intestinal Syphilis.** Revista De La Asociación Médica Argentina, January, February and March, 1915. Drs. Mariano R. Castex and Nicolás Romano report a case. The patient denied venereal disease. He had diarrhoea, 8-10 stools a day, abundant and filled with mucus, pus and blood. He complained of pain and tenderness in the left iliac region. There existed a bilateral optic neuritis. The proctosigmoidoscope revealed an intense hyperemia of the rectum and sigmoid with numerous ulcers, some with a hemorrhagic base and others necrotic. Lavage with nitrate of silver solution for 25 days had no effect. They eliminated dysentery, gonorrhoea, carcinoma, tuberculosis, and ulcerative colitis, and instituted anti-syphilitic treatment:—iodide of potassium and intravenous injections of the cyanide of mercury. Three intravenous injections of salvarsan were given. A cure resulted. A Wassermann test after the anti-syphilitic treatment resulted negatively. These cases are rare and hard to diagnose clinically. They consider that cases are more frequent in children than in adults. Any part of the intestine may be affected but the large gut is the favorite site. Occlusion of the gut may occur from cicatricial contraction. They believe syphilis should be looked for in all rebellious cases of diarrhoea accompanied by mucus and pus. If not interrupted by apropriate treatment, death results. Too often they are diagnosed post-mortem. The prognosis depends on the diagnosis.

